# Explicitly Slow, Implicitly Fast, or the Other Way Around? Brain Mechanisms for Word Acquisition

**DOI:** 10.3389/fnhum.2019.00116

**Published:** 2019-04-26

**Authors:** Yury Shtyrov, Alexander Kirsanov, Olga Shcherbakova

**Affiliations:** ^1^Department of Clinical Medicine, Center of Functionally Integrative Neuroscience, Aarhus University, Aarhus, Denmark; ^2^Laboratory of Behavioural Neurodynamics, Saint Petersburg State University, Saint Petersburg, Russia; ^3^Department of General Psychology, Faculty of Psychology, Saint Petersburg State University, Saint Petersburg, Russia

**Keywords:** learning, memory, fast mapping, word, brain, neocortex, neuroimaging, explicit encoding

Our ability to communicate using language is a specific cognitive faculty that makes humans stand apart from all other animal species on the planet. Despite the crucial role that language plays in our individual and social well-being, the origins of language are still poorly understood from both evolutionary and ontogenetic perspectives. One of the key gaps in the knowledge lies in the understanding of specific cognitive and neural bases of language acquisition that underpin our successful and efficient ability to learn a large number of new words, both as children at all stages of development and as adults when learning a new language or novel professional lexicon. This opinion paper briefly overviews the main systems involved in word acquisition, identifies gaps in the existing evidence and suggests possible ways to close them.

The behavioral and neural mechanisms of word acquisition remain a debated topic (for reviews, see e.g., Dollaghan, 1985; Davis and Gaskell, 2009). On the systems level, learning processes are most commonly separated into initial encoding and later consolidation. The stage of encoding is believed to occur rapidly and to involve multiple brain areas, most crucially medial temporal lobe (MTL) including hippocampus and parahippocampal cortices (McClelland et al., 1995; Suzuki, 2006); the consolidation, in turn, is a more gradual process leading to the formation of long-term memory traces in the neocortex (Walker and Stickgold, 2006; Battaglia et al., 2011). Such a two-stage or “complementary learning systems” approach resonates through different levels of investigations, including animal studies with hippocampal and cortical lesions trying to disentangle the two stages (Talpos et al., 2008), cognitive science models using computational neural networks to simulate neural memory build-up processes (O'Reilly and McClelland, 1994), as well as patient studies using hippocampally-damaged amnesiacs that demonstrate specific patterns of retrograde memory loss (Scoville and Milner, 1957; Sharon et al., 2011). A range of experiments extended this approach to account for the brain's word learning mechanisms, with their results indicating that newly-learnt word-forms fully enter the lexicon only after an overnight consolidation period, which is accompanied by changes in neocortical and MTL activity (Gaskell and Dumay, 2003; Davis and Gaskell, 2009). While this framework can successfully explain a range of phenomena in the fields of memory, learning, and language, another body of observations suggests the existence of a hippocampally independent route for direct acquisition of new word forms by the neocortex, at least under certain conditions (Shtyrov, 2012), as we will discuss below.

Whereas different processes (e.g., imitation, repetition, association, or generalization) may be involved in learning, the initial acquisition of new words in real-life situations can arguably be achieved through two main learning strategies: a direct explicit instruction (e.g., “This is a glorp, please remember it”) or a contextually-driven implicit inference/deduction (“There is a toy car, a book and a glorp on the table. Which color is the glorp?”). Although not mutually exclusive, these two are characterized by dissociable (to a degree at least) properties. Explicit learning, often dubbed explicit encoding (EE), is usually associated with repetitive presentation occurring over extended (or even multiple) practice sessions, such as classroom instruction or rehearsal. In contrast, contextually-driven deduction normally takes place in routine daily interactions between individuals and appears to have a near-immediate effect, evident before long-term memory consolidation processes set in. For building up new semantic representations, it requires just a few expositions (with claims of even single-shot learning) in a context that facilitates inference through exclusion or deduction (Bloom and Markson, 1998; Halberda, 2006; Horst and Samuelson, 2008). This rapid implicit acquisition is often termed fast mapping (FM) and is considered to be a general learning mechanism that plays a key role in acquiring new words and their semantics in the process of natural language learning (Carey and Bartlett, 1978; Kaminski et al., 2004).

Even though, as discussed below, teasing the two mechanisms apart is not straightforward, it is this latter strategy, FM, which has been argued to predominantly depend on the neocortex and be largely independent from the MTL and hippocampo-neocortical consolidation circuits. FM appears to be most efficient in children, in whom hippocampus and episodic memory are not fully developed (Bauer, 2008). Clinical investigations in patients with MTL lesions have shown that explicit exposure to new information results in poor behavioral outcomes, while FM learning regimes, on the one hand, lead to successful acquisition, and, on the other hand, are hampered by neocortical damage (Sharon et al., 2011; Warren and Duff, 2014). BOLD-fMRI studies in healthy adults show that FM, in comparison to EE tasks, activates a more widespread neocortical network during encoding, which seems to most reliably include the anterior-temporal lobe, ATL (Atir-Sharon et al., 2015; Merhav et al., 2015). Left ATL neocortex, in turn, has been repeatedly suggested as a seat of lexico-semantic representations, playing the role of a central “hub” in distributed word memory circuits (Patterson et al., 2007). Furthermore, while EE seems to benefit from an overnight consolidation stage, learning via fast mapping does not trigger overnight changes in brain representations (Merhav et al., 2015). Moreover, even passive exposure to unattended novel word forms presented repeatedly outside of any task or context leads to immediate changes in the brain responses, indicative of a novel memory trace build-up in the perisylvian neocortex (Kimppa et al., 2015, 2016; Partanen et al., 2017, 2018), provided the exposure is intensive enough (dozens to hundreds of repetitions). Such different brain signatures of the two learning strategies in themselves support (partially) different mechanisms underpinning them and may thus explain diverging learning dynamics and efficiency. In sum, even though in real-life situations the distinctions between the two strategies may be blurred, with both mechanisms at play simultaneously depending on the context and the learning environment, the available evidence allows to conclude that they can be dissociated at the conceptual level as well as behaviorally and neurophysiologically.

However, these findings still leave a number of questions open. First, findings of any advantages offered by FM and/or differential learning outcomes of the two regimes have been questioned by some studies that failed to replicate them (see, e.g., Greve et al., 2014). On the other hand, in spite of frequent claims of FM benefits for learning, most of the above studies in fact show better recognition rates after EE (although this does not *per se* undermine the distinctions found between the brain mechanisms). Second, the behavioral routines typically used to contrast the learning regimes differ in more than one dimension. The most typical paradigm used to implement this (see e.g., Merhav et al., 2015) uses a word-picture association approach, in which the FM condition presents the subject with two or more images, only one them being novel, thus requiring inference to understand which of the objects the new word refers to (e.g., “does the glorp have leaves?”); at the same time, the EE condition often presents only a single image in conjunction with its name (“this is a glorp”). Such a design implies a lack of basic visual balancing between the two conditions, which puts differential load already at the level of initial visual processing of the stimuli. Furthermore, at the higher cognitive level, it creates different distribution of attention across the visual field between the two conditions. Whereas attention and executive control can certainly influence learning outcomes (Kimppa et al., 2016), it is important to disentangle their effects and those more directly related to memory or language systems as such.

Third, while these two conditions inevitably frame the task in cognitively different manners, it is further exacerbated by the way the instruction is typically offered in such an experiment. In FM condition (Carey and Bartlett, 1978; Atir-Sharon et al., 2015), a question (“does the glorp have leaves?”) or a request (“bring/show me the glorp”) are used, whereas naming is used in EE (“this is a glorp”). Pragmatically, Naming, Question and Request constitute different speech acts (Searle, 1969) that put different demands on the cognitive system and are known to be underpinned by overlapping yet distinct brain networks (van Ackeren et al., 2012; Egorova et al., 2014), which further confounds any behavioral and neurophysiological distinctions found between FM and EE. While it may not be possible to fully balance the two clearly distinct learning regimes, minimizing the effects of any extraneous factors, such as visual features, attention, cognitive load, and contextual framing, it is highly desirable to disentangle their mechanisms with fewer confounding factors.

More generally, studies diverge hugely in how they train their subjects with new words. This could be word-picture associations that use written or spoken forms or both modalities (Breitenstein et al., 2005), purely sentential context (Mestres-Missé et al., 2007, 2008) or even isolated word forms with no semantics (Gaskell and Dumay, 2003; Shtyrov et al., 2010). Some of the studies use perceptual exposure, while others introduce articulation as an ecological part of the learning process (Rauschecker et al., 2008). Similar to the points above, different learning modalities would introduce the variability into results, complicating the overall picture. Direct comparisons of visual vs. auditory mode of acquisition (the latter being the “native” modality of language), learning in vs. outside context, with vs. without semantic reference, perceptually only vs. with articulation etc. would be important to disentangle all of these factors.

Equally important is the assessment of the learning outcomes. The tasks used for this diverge across studies, and most often include free recall, lexical decision and familiarity judgement. These are more shallow lexical tasks, which may not require full lexico-semantic access of the newly formed memory trace. A more elaborate testing that could require lexical as well as semantic (e.g., semantic judgement task, semantic matching, free-form definition), and possibly even contextual levels of testing, would therefore be desirable. Further, the assessment of semantics acquisition could also be done on the basis of brain activation patterns, such as the recruitment of meaning-dependant modality-specific networks (Macedonia et al., 2011; Vukovic and Shtyrov, 2014; Mayer et al., 2015).

On a similar note, many studies limit themselves to immediate post-experimental testing, ignoring the longer-term consolidation processes that play a significant role in (at least some types) of acquisition (McMurray et al., 2016). Ideally, the assessment of the learning outcomes should be done both immediately and after an overnight sleep period; longer-term retention of stimulus materials over weeks/months could also be addressed where possible.

Finally, and importantly, the bulk of previous research in this area was done behaviorally and/or using slow neuroimaging tools, such as fMRI, to address distinctions between learning regimes. As such, these measures cannot address rapid neuronal activations that are known to take place on the millisecond range; this is particularly important for language, a function that relies on temporally dynamic processing of information rapidly unfolding over time (Friederici, 2002; Pulvermüller et al., 2009; MacGregor et al., 2012; Shtyrov and Stroganova, 2015). To better understand the neural processes underpinning different types of language learning, there is a need for a more direct measure of electric neuronal activity, which can be provided by time-resolved imaging tools such as EEG or MEG, or, ideally a combination of tools, such as MRI-based source analysis of combined multichannel EEG-MEG data. On the flip-side, while activity patterns obtained in brain studies are useful, causal evidence is also needed to scrutinize these distinctions in healthy individuals. Outside of limited patient studies, such evidence is presently lacking. The use of targeted neurostimulation techniques (such as TMS or tDCS) to influence the learning outcomes may provide the much needed evidence for the involvement of particular brain areas in specific learning types.

On a more conceptual level, the use of learning strategies might differ according to the learning environment, resources, and purposes, while their effectiveness may also vary depending on the learner's age, neural development, cognitive capacities, and overall context. Furthermore, in the natural language acquisition scenario (other than classroom settings), word acquisition, whether in the first or second language, is unlikely to occur exclusively through only one or the other strategy. Instead, both strategies may be used concurrently which may possibly result in enhanced learning outcomes, although the extent to which each strategy is used depends on the learning environment and the language (first or second) in question. Notably, the brain networks implicated in the two mechanisms do overlap (most importantly in the temporal lobe) and the tight connectivity, which is known to exist between these structures (Catani and Mesulam, 2008; Friederici, 2012), provides for seamless information exchange across the circuits involved. Furthermore, a range of other processes involved in learning (e.g., association, differentiation, enrichment, retrieval) may interactively influence the acquisition of new materials at different stages. Finally, the explicit/implicit distinction is also present in more general models of language, not just word acquisition (e.g., Ullman, 2001; Paradis, 2009). These and similar factors should also be considered in studies investigating learning strategies.

To conclude, the literature to date clearly suggests overlapping yet dissociable learning systems that support different routes of novel word acquisition by the human brain. They diverge in their speed and underlying brain structures, and may be used to different extents for explicitly acquiring presented information or for contextually-driven implicit inference-based learning. The studies available to date diverge in the methodologies employed and present a somewhat controversial picture. To fill these gaps in the field, future studies should use a combination of rigorously matched behavioral regimes, controlled modes of presentation, a comprehensive set of tasks to assess the outcomes at different times, and different neuroimaging tools able to assess both the complex spatio-temporal dynamics of word acquisition, and the causal relationships between brain structures and learning strategies.

## Author Contributions

YS conceived the original idea for the paper. All authors wrote and revised the manuscript and have approved the content for publication.

### Conflict of Interest Statement

The authors declare that the research was conducted in the absence of any commercial or financial relationships that could be construed as a potential conflict of interest.

## References

[B1] Atir-Sharon T. Gilboa A. Hazan H. Koilis E. Manevitz L. M. (2015). Decoding the formation of new semantics: MVPA investigation of rapid neocortical plasticity during associative encoding through fast mapping. Neural Plasticity 2015:804385. 10.1155/2015/804385 26257961 PMC4519547

[B2] Battaglia F. P. Benchenane K. Sirota A. Pennartz C. M. Wiener S. I. (2011). The hippocampus: hub of brain network communication for memory. Trends Cogn. Sci. 15, 310–318. 10.1016/j.tics.2011.05.008 21696996

[B3] Bauer P. J. (2008). Toward a neuro-developmental account of the development of declarative memory. Dev. Psychobiol. 50, 19–31. 10.1002/dev.20265 18085555

[B4] Bloom P. Markson L. (1998). Capacities underlying world learning. Trends Cogn. Sci. 2, 67–73. 10.1016/S1364-6613(98)01121-8 21227068

[B5] Breitenstein C. Jansen A. Deppe M. Foerster A. F. Sommer J. Wolbers T.. (2005). Hippocampus activity differentiates good from poor learners of a novel lexicon. NeuroImage 25, 958–968. 10.1016/j.neuroimage.2004.12.019 15808996

[B6] Carey S. Bartlett E. (1978). Acquiring a single new word, in Papers and Reports on Child Language Development, 17–29. Retrieved from: http://www.mendeley.com/research/acquiring-single-new-word-1/ (accessed February 28, 2019).

[B7] Catani M. Mesulam M. (2008). The arcuate fasciculus and the disconnection theme in language and aphasia: history and current state. Cortex 44, 953–961. 10.1016/j.cortex.2008.04.002 18614162 PMC2740371

[B8] Davis M. H. Gaskell M. G. (2009). A complementary systems account of word learning: neural and behavioural evidence. Phil. Trans. R. Soc. B. 364, 3773–3800. 10.1098/rstb.2009.0111 19933145 PMC2846311

[B9] Dollaghan C. (1985). Child meets word: “fast mapping” in preschool children. J. Speech Hear. Res. 28, 449–454. 10.1044/jshr.2803.454 4046586

[B10] Egorova N. Pulvermüller F. Shtyrov Y. (2014). Neural dynamics of speech act comprehension: an MEG study of naming and requesting. Brain Topogr. 27, 375–392. 10.1007/s10548-013-0329-3 24253730 PMC3976511

[B11] Friederici A. D. (2002). Towards a neural basis of auditory sentence processing. Trends Cogn. Sci. 6, 78–84. 10.1016/S1364-6613(00)01839-8 15866191

[B12] Friederici A. D. (2012). The cortical language circuit: from auditory perception to sentence comprehension. Trends Cogn. Sci. 16, 262–268. 10.1016/j.tics.2012.04.001 22516238

[B13] Gaskell M. G. Dumay N. (2003). Lexical competition and the acquisition of novel words. Cognition 89, 105–132. 10.1016/S0010-0277(03)00070-2 12915296

[B14] Greve A. Cooper E. Henson R. N. (2014). No evidence that “fast-mapping” benefits novel learning in healthy Older adults. Neuropsychologia 60, 52–59. 10.1016/j.neuropsychologia.2014.05.011 24939237 PMC4115174

[B15] Halberda J. (2006). Is this a dax which I see before me? Use of the logical argument disjunctive syllogism supports word-learning in children and adults. Cogn. Psychol. 53, 310–344. 10.1016/j.cogpsych.2006.04.003 16875685

[B16] Horst J. S. Samuelson L. K. (2008). Fast mapping but poor retention by 24-month-old infants. Infancy 13, 128–157. 10.1080/15250000701795598 33412722

[B17] Kaminski J. Call J. Fischer J. (2004). Word learning in a domestic dog: evidence for “fast mapping.” Science 304, 1682–1683. 10.1126/science.1097859 15192233

[B18] Kimppa L. Kujala T. Leminen A. Vainio M. Shtyrov Y. (2015). Rapid and automatic speech-specific learning mechanism in human neocortex. NeuroImage 118, 282–291. 10.1016/j.neuroimage.2015.05.098 26074199

[B19] Kimppa L. Kujala T. Shtyrov Y. (2016). Individual language experience modulates rapid formation of cortical memory circuits for novel words. Sci. Rep. 6, 1–10. 10.1038/srep30227 27444206 PMC4957205

[B20] Macedonia M. Müller K. Friederici A. D. (2011). The impact of iconic gestures on foreign language word learning and its neural substrate. Hum. Brain Mapp. 32, 982–998. 10.1002/hbm.21084 20645312 PMC6870319

[B21] MacGregor L. J. Pulvermüller F. Van Casteren M. Shtyrov Y. (2012). Ultra-rapid access to words in the brain. Nat. Commun. 3:711. 10.1038/ncomms1715 22426232 PMC3543931

[B22] Mayer K. M. Yildiz I. B. Macedonia M. von Kriegstein K. (2015). Visual and motor cortices differentially support the translation of foreign language words. Curr. Biol. 25, 530–535. 10.1016/j.cub.2014.11.068 25660537

[B23] McClelland J. L. McNaughton B. L. O'Reilly R. C. (1995). Why there are complementary learning systems in the hippocampus and neocortex: Insights from the successes and failures of connectionist models of learning and memory. Psychol. Rev. 102, 419–457. 10.1037/0033-295X.102.3.419 7624455

[B24] McMurray B. Kapnoula E. Gaskell M. G. (2016). Learning and integration of new wordforms: Consolidation, pruning and the emergence of automaticity, in Speech Perception and Spoken Word Recognition, eds Gaskell M. G. Mirkovic J. (London: Taylor and Francis), 116–142. 10.4324/9781315772110

[B25] Merhav M. Karni A. Gilboa A. (2015). Not all declarative memories are created equal: fast mapping as a direct route to cortical declarative representations. NeuroImage 117, 80–92. 10.1016/j.neuroimage.2015.05.027 25988227

[B26] Mestres-Missé A. Càmara E. Rodriguez-Fornells A. Rotte M. Münte T. F. (2008). Functional neuroanatomy of meaning acquisition from context. J. Cogn. Neurosci. 20, 2153–2166. 10.1162/jocn.2008.20150 18457509

[B27] Mestres-Missé A. Rodriguez-Fornells A. Münte T. F. (2007). Watching the brain during meaning acquisition. Cerebral Cortex 17, 1858–1866. 10.1093/cercor/bhl094 17056648

[B28] O'Reilly R. C. McClelland J. L. (1994). Hippocampal conjunctive encoding, storage, and recall: avoiding a trade-off. Hippocampus. 10.1002/hipo.450040605 7704110

[B29] Paradis M. (2009). Declarative and Procedural Determinants of Second Languages. Amsterdam: John Benjamins.

[B30] Partanen E. Leminen A. de Paoli S. Bundgaard A. Kingo O. S. Krøjgaard P.. (2017). Flexible, rapid and automatic neocortical word form acquisition mechanism in children as revealed by neuromagnetic brain response dynamics. NeuroImage 155, 450–459. 10.1016/j.neuroimage.2017.03.066 28389383

[B31] Partanen E. J. Leminen A. Cook C. Shtyrov Y. (2018). Formation of neocortical memory circuits for unattended written word forms: neuromagnetic evidence. Sci. Rep. 8:15829. 10.1038/s41598-018-34029-y 30361630 PMC6202413

[B32] Patterson K. Nestor P. J. Rogers T. T. (2007). Where do you know what you know? The representation of semantic knowledge in the human brain. Nat. Rev. Neurosci. 8, 976–987. 10.1038/nrn2277 18026167

[B33] Pulvermüller F. Shtyrov Y. Hauk O. (2009). Understanding in an instant: neurophysiological evidence for mechanistic language circuits in the brain. Brain Lang. 110, 81–94. 10.1016/j.bandl.2008.12.001 19664815 PMC2734884

[B34] Rauschecker A. M. Pringle A. Watkins K. E. (2008). Changes in neural activity associated with learning to articulate novel auditory pseudowords by covert repetition. Hum. Brain Mapp. 29, 1231–1242. 10.1002/hbm.20460 17948887 PMC6870739

[B35] Scoville W. B. Milner B. (1957). Loss of recent memory after bilateral hippocampal lesions. J. Neurol. Neurosurg. Psychiatry 20, 11–21. 10.1136/jnnp.20.1.11 13406589 PMC497229

[B36] Searle J. R. (1969). Speech Acts: An Essay in the Philosophy of Language. Cambridge: Cambridge University Press.

[B37] Sharon T. Moscovitch M. Gilboa A. (2011). Rapid neocortical acquisition of long-term arbitrary associations independent of the hippocampus. Proc. Natl. Acad. Sci. 108, 1146–1151. 10.1073/pnas.1005238108 21199935 PMC3024695

[B38] Shtyrov Y. (2012). Neural bases of rapid word learning. Neuroscientist 18, 312–319. 10.1177/1073858411420299 22020546

[B39] Shtyrov Y. Nikulin V. V. Pulvermüller F. (2010). Rapid cortical plasticity underlying novel word learning. J. Neurosci. 30, 16864–16867. 10.1523/JNEUROSCI.1376-10.2010 21159957 PMC6634920

[B40] Shtyrov Y. Y. Stroganova T. A. (2015). When ultrarapid is ultrarapid: on importance of temporal precision in neuroscience of language. Front. Hum. Neurosci. 9, 1–5. 10.3389/fnhum.2015.00576 26539098 PMC4612669

[B41] Suzuki W. A. (2006). Encoding new episodes and making them stick. Neuron 50, 19–21. 10.1016/j.neuron.2006.03.029 16600852

[B42] Talpos J. C. Dias R. Bussey T. J. Saksida L. M. (2008). Hippocampal lesions in rats impair learning and memory for locations on a touch-sensitive computer screen: the “ASAT” task. Behav. Brain Res. 192, 216–225. 10.1016/j.bbr.2008.04.008 18499279

[B43] Ullman M. T. (2001). A neurocognitive perspective on language: the declarative/procedural model. Nat Rev Neurosci 2, 717–726. 10.1038/35094573 11584309

[B44] van Ackeren M. J. Casasanto D. Bekkering H. Hagoort P. Rueschemeyer S. A. (2012). Pragmatics in action: indirect requests engage theory of mind areas and the cortical motor network. J. Cogn. Neurosci. 24, 2237–47. 10.1162/jocn_a_00274 22849399

[B45] Vukovic N. Shtyrov Y. (2014). Cortical motor systems are involved in second- language comprehension: evidence from rapid mu-rhythm desynchronisation. NeuroImage 102, 695–703. 10.1016/j.neuroimage.2014.08.039 25175538

[B46] Walker M. P. Stickgold R. (2006). Sleep, memory, and plasticity. Ann. Rev. Psychol. 57, 139–166. 10.1146/annurev.psych.56.091103.070307 16318592

[B47] Warren D. E. Duff M. C. (2014). Not so fast: Hippocampal amnesia slows word learning despite successful fast mapping. Hippocampus 24, 920–933. 10.1002/hipo.22279 24719218 PMC4301585

